# Accuracy and fairness of facial recognition technology in real-world, low-quality police images: An experiment

**DOI:** 10.1371/journal.pone.0353793

**Published:** 2026-10-21

**Authors:** Maria Cuellar, Hon Kiu (James) To, Arush Mehrotra

**Affiliations:** 1 Department of Criminology, University of Pennsylvania, Philadelphia, Pennsylvania, United States of America; 2 Department of Statistics and Data Science, Wharton School, University of Pennsylvania, Philadelphia, Pennsylvania, United States of America; 3 University of Pennsylvania, School of Engineering and Applied Science, Philadelphia, Pennsylvania, United States of America; Florida International University, UNITED STATES OF AMERICA

## Abstract

Facial recognition technology (FRT) is increasingly used in criminal investigations, yet most evaluations of its accuracy rely on high-quality images, unlike those often encountered by law enforcement. This study examines how five common forms of image degradation (contrast, brightness, motion blur, pose shift, and resolution) affect FRT accuracy, and how that varies by gender and race. Using synthetic faces generated by StyleGAN3 and labeled with FairFace, we simulate degraded images and evaluate performance using the DeepFace framework with the ArcFace model in 1:n identification tasks. By using simulated faces, we are able to estimate conditional FPIR and FNIR error-rate curves across degradation levels under controlled conditions, providing practical guidance on how database search performance changes as questioned image quality deteriorates. We find that high motion blur and contrast and low resolution and brightness substantially increase FNIR. At the selected operating point, FPIR is substantially higher than FNIR and is highest near baseline image quality, generally decreasing as image degradation increases and demographic disparities persist across degradation conditions. Demographic disparities vary by error type: FPIR is often higher for Black individuals, particularly Black women, whereas the demographic groups with the highest FNIR differ across degradation conditions. These disparities raise concerns about fairness and reliability of the use of FRT. These findings highlight the importance of evaluating algorithmic performance using the low-quality images encountered in criminal investigations to ensure both fairness and validity.

## 1 Introduction

Law enforcement agencies increasingly use facial recognition technology (FRT) to identify suspects, locate missing persons, and generate investigative leads [1]. In a typical 1:n identification workflow, a probe image is searched against a database and the system returns candidate images for human review; recent work suggests that newer algorithms may make this review stage more difficult by returning more visually plausible nonmatches [2]. Despite increasing deployment, recent reviews conclude that empirical evidence about the operational effectiveness and equity of FRT in policing remains limited [1]. As law enforcement gains access to increasing quantities of images collected under uncontrolled conditions, understanding how FRT performs on low-quality images becomes increasingly important.

Although FRT is increasingly used, and often promoted as highly accurate ([3]), the accuracy of FRTs has been tested primarily in settings with high-quality images ([4]) or in-the-wild image datasets, which contain uncontrolled variation but do not isolate the effects of specific image degradations. Algorithms likely work less well in low-quality images such as those often used by law enforcement. But, the promotion of FRTs being so accurate can lead to law enforcement being misled. For example, a media investigation revealed that some law enforcement officers treat FRT outputs as definitive rather than investigative leads, for example, referring to an unverified match as ‘100% match’ ([5]). However, the Innocence Project reports that there are “at least seven confirmed cases of misidentification due to facial recognition technology, six of which involve Black people who have been wrongfully accused.” ([6]).

But how often are FRTs making errors with degraded images, and how much worse are errors due to blur than to a face that is turned away? Are errors occurring equally across demographic groups? Without proper validation, using FRT in practice risks contributing to wrongful accusations or wrongful convictions, and this could be worse for individuals of certain demographic characteristics. Law enforcement officers and other stakeholders might trust the FRT results more or less than they should.

Prior work has evaluated the performance of both facial recognition algorithms and human facial comparison examiners under controlled conditions ([7]). A growing literature has also examined how image quality influences FRT accuracy [8–13], including the effects of object motion, camera characteristics, illumination, pose, and resolution. NIST has evaluated large numbers of facial recognition algorithms in both 1:1 and 1:n tasks and across demographic groups ([4]). More recent studies have directly examined the intersection of image degradation, 1:n identification, and demographic disparities. Atzori et al. documented demographic disparities under low-resolution conditions, although their magnitude and relationship to operating thresholds varied across models and datasets [14], while Pangelinan et al. found that increasing blur and reducing resolution can sharply degrade 1:n identification performance and alter demographic disparities [15]. More recent work by Pangelinan et al. showed that degrading both probe and gallery images can substantially increase FPIR and demographic disparities, with nonlinear interactions between probe quality, gallery quality, and the matching algorithm [16].

Differential accuracy of FRT across demographic groups remains a critical concern. Prior studies have documented differences in error rates by race, gender, and age [17–20], and different methods have been proposed for measuring algorithmic fairness [21–24]. Existing work therefore demonstrates that image quality and demographic characteristics can interact in determining FRT performance. However, relatively little work has systematically varied multiple forms of image degradation over a range of severity levels while separately estimating false positive and false negative identification rates under controlled conditions.

Courts have begun to require disclosure when a lead was generated using FRT, and it is likely that FRT results will increasingly be introduced in evidentiary contexts. As of early 2025, 15 states had enacted laws regulating FRT use in policing ([25]), and courts are beginning to require disclosure when FRT is used. As such, courts’ admissibility standards for scientific evidence offer a valuable framework for evaluating the validity of these tools. Legal scholars have raised concerns about transparency, proprietary algorithms, and contextual bias [26–30]. As FRT moves closer to evidentiary use, its validity, reliability, and error rates will require careful empirical evaluation ([31]).

Our approach draws on lessons learned from problems in traditional forensic science disciplines, specifically, their lack of demonstrated reliability (accuracy, repeatability, and reproducibility) ([31]). It applies the corresponding solutions proposed in the forensic science reform literature ([32]) to the study of facial recognition technologies (FRTs).

Direct comparisons of reported error rates across forensic disciplines should be interpreted cautiously because studies often involve different tasks, populations, image qualities, protocols, and decision frameworks. Human examiner studies and algorithmic benchmark studies may also differ substantially in design and operational conditions. Accordingly, the goal of this study is not to compare FRT to other forensic disciplines, but rather to evaluate how image degradation affects FRT performance under controlled experimental conditions. The effects of image degradation on facial identification are also connected to the broader human perceptual literature. Prior work in psychology has shown that distance, resolution, and visual detail substantially affect the ability of humans to recognize faces ([33]), motivating the importance of systematically evaluating how image quality influences modern FRT systems.

In this study, we evaluate FRT performance by isolating the effects of image quality on accuracy and fairness. Using synthetic faces permits controlled experimental manipulation of image characteristics such as contrast, brightness, motion blur, pose, and resolution while maintaining known ground truth identities. Synthetic datasets also permit the generation of conditional error-rate curves across varying image conditions and demographic groups [34–36]. Because FRT systems are frequently deployed on low-quality surveillance images, understanding how performance changes under degraded conditions is important for evaluating both reliability and fairness in real-world criminal investigations.

## 2 Materials and methods

### 2.1 Synthetic face generation

To assess the impact of image quality on FRT performance, we generate a dataset of synthetic faces, following the process outlined in Fig 1. Synthetic data allows for precise experimental manipulation of image quality while eliminating confounding variables associated with real-world datasets. It is also a way to rotate the face’s pose experimentally for a large number of faces at once. The dataset consists of a “control” set, which includes high-quality images, and several “treatment” sets where images have been degraded in specific ways to simulate real-world conditions. The treatment images include variations in contrast, brightness, motion blur, pose, and resolution, allowing for a detailed analysis of how these factors influence FRT accuracy.

**Fig 1:**
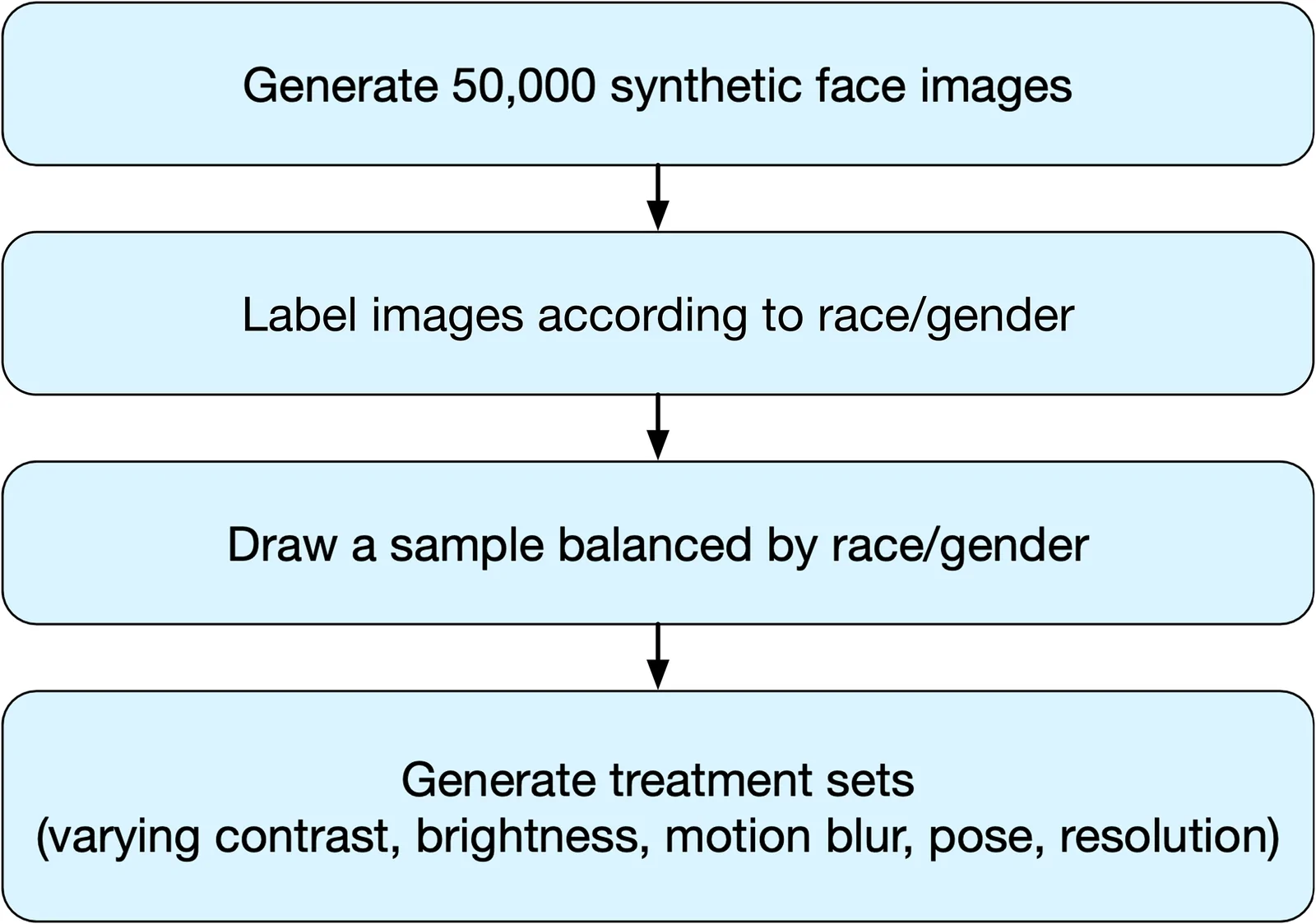
Data generation procedure. We generate a large population of synthetic faces, and then take a random sample that is balanced by race and gender. We then use this to generate treatment and control groups.

A state-of-the-art facial recognition algorithm was used to compare synthetic images against a database, measuring both false positive and false negative rates. The false positives are instances for which the algorithm incorrectly identifies an individual as a match, while the false negatives are instances for which the algorithm incorrectly rejects a match. Error rates were analyzed across different racial and gender groups to determine whether FRT exhibits systematic biases that could affect certain populations.

We first generate base synthetic faces using StyleGAN3 ([37]). StyleGAN3 is a popular face generation model that is capable of generating high-quality, photo-realistic faces ([38,39]). Users can freely choose their desired pre-trained network for generation, as well as a truncation parameter 0≤ψ≤1 ([40,41]). The pre-trained network is trained on a training dataset (i.e., the set of faces used to train the network and allow it to learn the features and patterns of the faces) for face generation, and the truncation parameter ψ controls the degree of weighting away from the “average” face of the pre-trained network. In particular, choosing ψ=0 always returns the “average” face of the pre-trained network. The truncation parameter ψ also plays a role in facial variety. A smaller ψ tends to reduce the facial variety of the generated faces (see, e.g., [40–42]). Some common choices of ψ are 0.5 and 0.7.

For this study, we select the pre-trained network based on the Flickr-Faces-HQ (FFHQ) dataset, which consists of 70,000 high-quality PNG images at 1024×1024 resolution and contains considerable variation in terms of age, ethnicity and image background. It also includes photos with accessories such as eyeglasses, sunglasses, hats, etc. The images were crawled from Flickr, thus inheriting all the biases of that website, and automatically aligned and cropped using dlib. A truncation parameter of ψ=0.7 was used ([40]). Fig 2 shows some examples of faces generated with StyleGAN3 based on the FFHQ network and a truncation parameter of ψ=0.7, and classified using FairFace in terms of gender and three races. Utilizing the StyleGAN3 algorithm, we generate an initial database consisting of 50,000 faces.

**Fig 2:**
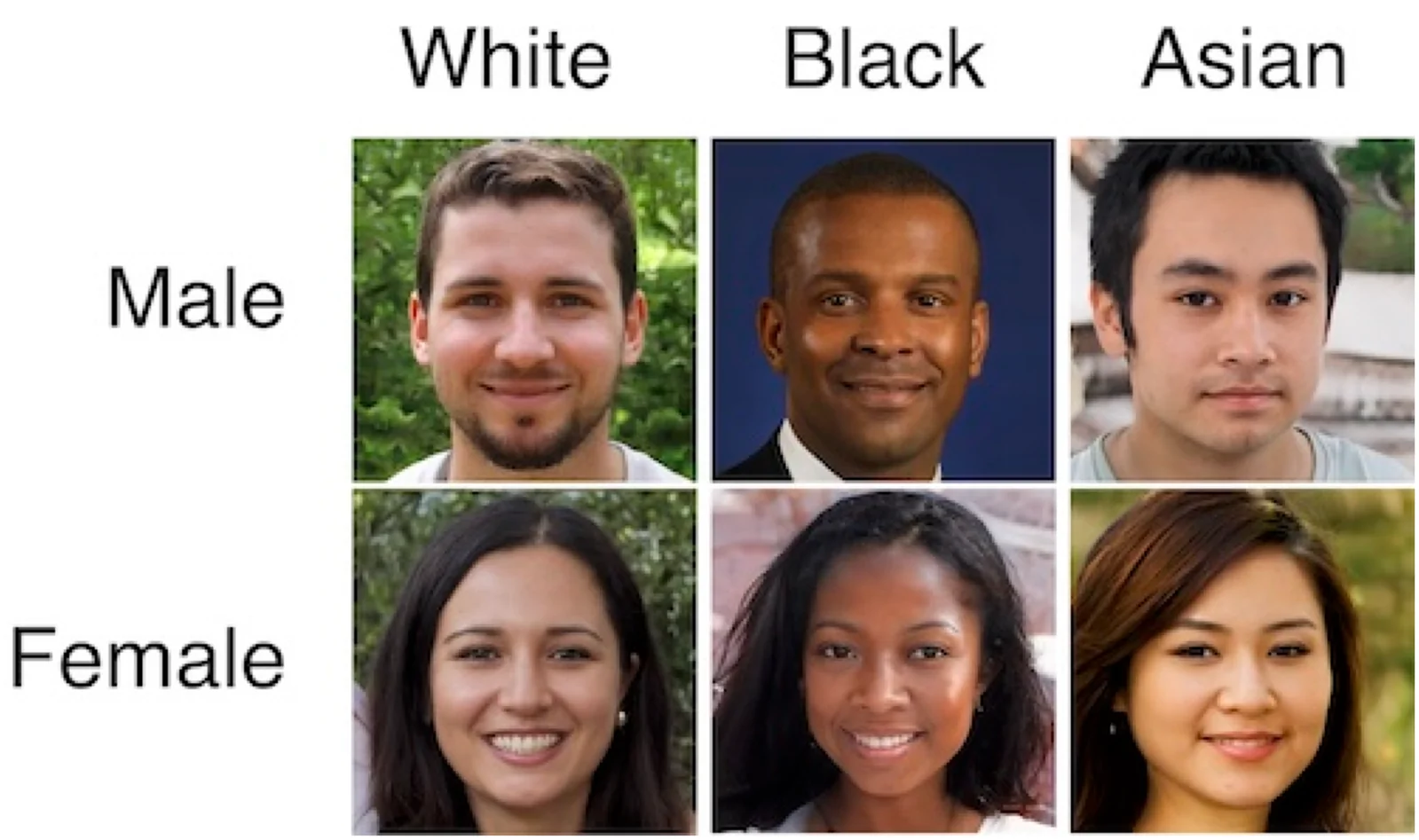
Examples of synthetic faces generated with StyleGAN3 and labeled with FairFace. We provide very basic gender and race features as a simplification to explore the conditional error-rate curves for degraded images. Future work should provide more careful gender and racial seed labeling.

### 2.2 Sampling scheme

From the pool of 50,000 generated faces, we randomly sample 167 as the “control” database. The sampling is performed using stratified random sampling so that the control database is balanced across race and gender. The number of control faces, 167, is selected based on the MRMCsamplesize package ([43]), using the sampleSize_Standalone function with an expected sensitivity of 0.99, half-width 0.025 for confidence intervals, and an assumed power of 90%.

To understand how the size of the database affects the performance we refer to [44]. The authors find that as database size increases, both false positives and false negatives rise in target-present lineups, and false positives rise in target-absent lineups. Understanding how increasing database size affects the accuracy changes based on image degradation factors is outside the scope of this article and is left as future work.

We randomly sample 167 “treated” faces for comparison against the control database. The treated faces are sampled using the same stratified race-by-gender sampling procedure as the control database to achieve race-by-gender balance. In particular, the sampling is done so that 84 faces do not have their corresponding control faces in the database (target-absent), and the remaining 83 faces have their corresponding control faces in the database (target-present). Our sampling process gives a Monte Carlo approximation to a 95% confidence interval. This is an empirical confidence interval constructed via repeated sampling from the population, a process that we can do here because we have a large sample of synthetic data.

### 2.3 Race and gender sample distribution

We emphasize that race is not a biological category but a social construct that depends on history and context, is often assigned rather than self-identified, and is an imprecise and ethically sensitive variable, points that have been widely discussed in the criminology and statistics literature (e.g., [45–48]), among others. Some researchers have a more careful treatment of race in FRT than we do [49], and some note that there are ethical concerns about auditing a FRT algorithm since the audit itself might “exacerbate or complement the harms propagated by the audited system” [50]. Our use of race categories is not meant to endorse their validity as natural kinds, but rather to evaluate how facial recognition technology performs with respect to categories that are widely used, rightly or wrongly, in both benchmark datasets and real-world deployments. A more refined analysis would consider mixed-race identities, alternative constructions of race, and uncertainty in labels. An alternative approach is exemplified by NIST’s Face Recognition Vendor Test (FRVT), which avoids direct racial labels and instead analyzes accuracy using country-of-origin and passport information, reflecting a different operationalization of demographic grouping [17,51]. Future work should assess how alternative constructions of demographic variables affect measured disparities and conclusions about algorithmic performance and fairness.

With this in mind, we proceed with the purpose of understanding how one definition of race interacts with image degradation factors. We create a balanced sample of demographic features of the generated faces. To do this, we first assess the demographic features of our generated faces using FairFace, a facial attribute analysis tool. FairFace predicts the demographic labels, including races, age groups, and genders ([52]). The FairFace algorithm has been shown to have high predictive performance across race, gender, and age groups ([52]), and is used for labeling faces ([53]).

Regarding age, we remove all the children from our sample, who were labeled by FairFace as being under age 10. Age 10 is the minimum age of juvenile adjudication in 15 states and territories ([54]) or the minimum age of criminal responsibility in other places, such as Hong Kong ([55]), England and Wales ([56]), and South Australia ([57]). Regarding race, we adopt four races provided by FairFace (White, Asian, Black, and Indian), but we extract only Black, White, and Asian for simplicity. The original “Asian” and “Indian” are combined into one single “Asian” group. A more nuanced treatment of race (e.g., include more races and account for ethnicity) is important and left for future research. The faces are generated from a pre-trained network that utilizes the FFHQ dataset as training samples, so it inherits characteristics of the training dataset, which are reflected in the generated faces.

Our original sample is 73% white, 17% Asian, 5% Black, and 5% Indian, while the 2020 US Census ([58]) states that the US population is 57.8% white (non-Hispanic), 5.9% Asian, 12.1% Black, 0.7% American Indian and Alaska Native alone (non-Hispanic). The Census percentages reported here do not sum to 100% because we report only the racial categories selected for comparison. The gender distribution of the generated faces is well-balanced, and the distribution also closely resembles that of the United States, which has 50.4% females. We do not study age distributions here. We use stratified random sampling to construct control databases that are balanced across race and gender. Table 1 shows the demographic distributions of our synthetic face control sample.

**Table 1:** Summary statistics of the synthetic face dataset used to construct the experimental control samples. This is the overall summary by gender and race after dropping children, combining the selected races, and dropping problematic images that raise exceptions when editing their poses.

	Count	Percentage
**Gender**		
Female	22,191	49.937%
Male	22,247	50.063%
**Race**		
White	32,425	72.967%
Black	2,298	5.171%
Asian	9,715	21.862%
**Total cases**	44,438	100%

### 2.4 Image degradation factors

The next step is editing the generated faces to alter the image qualities at various levels. Fig 3 shows example images that have been degraded. We normalize the parameter values for all degradation factors (from -1.0 to 1.0, with 0 being the baseline, undegraded image) to do inter-factor comparisons. For this paper, we consider five factors: contrast, brightness, motion blur, pose, and resolution. We use Python’s cv2 module ([59]) for editing the brightness, contrast, and resolution, Python’s blurgenerator ([60]) module for generating motion blurs, and the InterFaceGAN module ([61]), implemented through the codes provided by [38] for editing the pose. Note that motion blur and resolution are only present above and below zero, respectively, because of the nature of the image degradation (we do not de-blur the original image further, and we do not try to get a better resolution than the original image’s). Appendix A provides more details about the parameters we used to generate the treatment groups.

**Fig 3:**
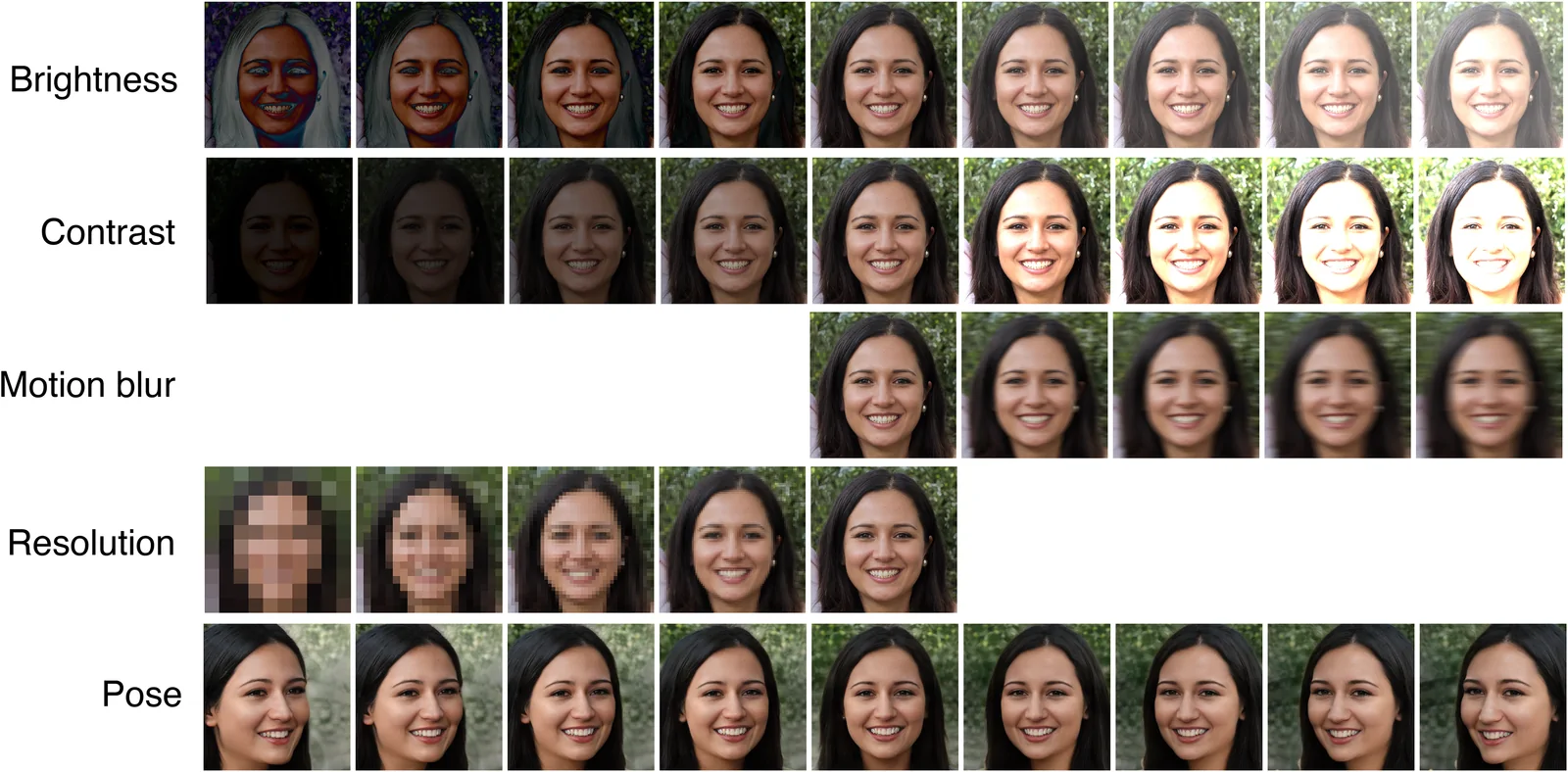
Facial images that have been modified according to parameters (brightness, contrast, motion blur, resolution, and pose). The image in the center is the baseline, control image.

## 3 Method

### 3.1 FRT algorithm

Our experimental design is outlined in Fig 4. The analyses were implemented in Python. As our FRT framework for evaluation, we selected DeepFace, a lightweight hybrid FRT framework that wraps the state-of-the-art facial recognition models ([62]). In particular, we chose the RetinaFace model ([63]) for face detection and the ArcFace model ([64]) for facial recognition, both of which were developed in the InsightFace project. ArcFace is recognized for its high accuracy and efficiency, and has been evaluated in NIST’s FRVT, where it stands out among open-source algorithms ([65,66]). The continued active development and strong empirical results ensure these models remain relevant and adaptable for future facial recognition challenges. To ensure that all “treated” images, including those with severe quality degradation, could be processed by the face recognition system, we set “enforce_detection = False” in DeepFace. This setting allows the system to compute embeddings even when automatic face detection fails, thereby preventing exceptions and enabling a comprehensive evaluation of performance under challenging conditions. While this approach may reduce accuracy, it reflects the need to assess system robustness with realistic, low-quality images.

**Fig 4:**
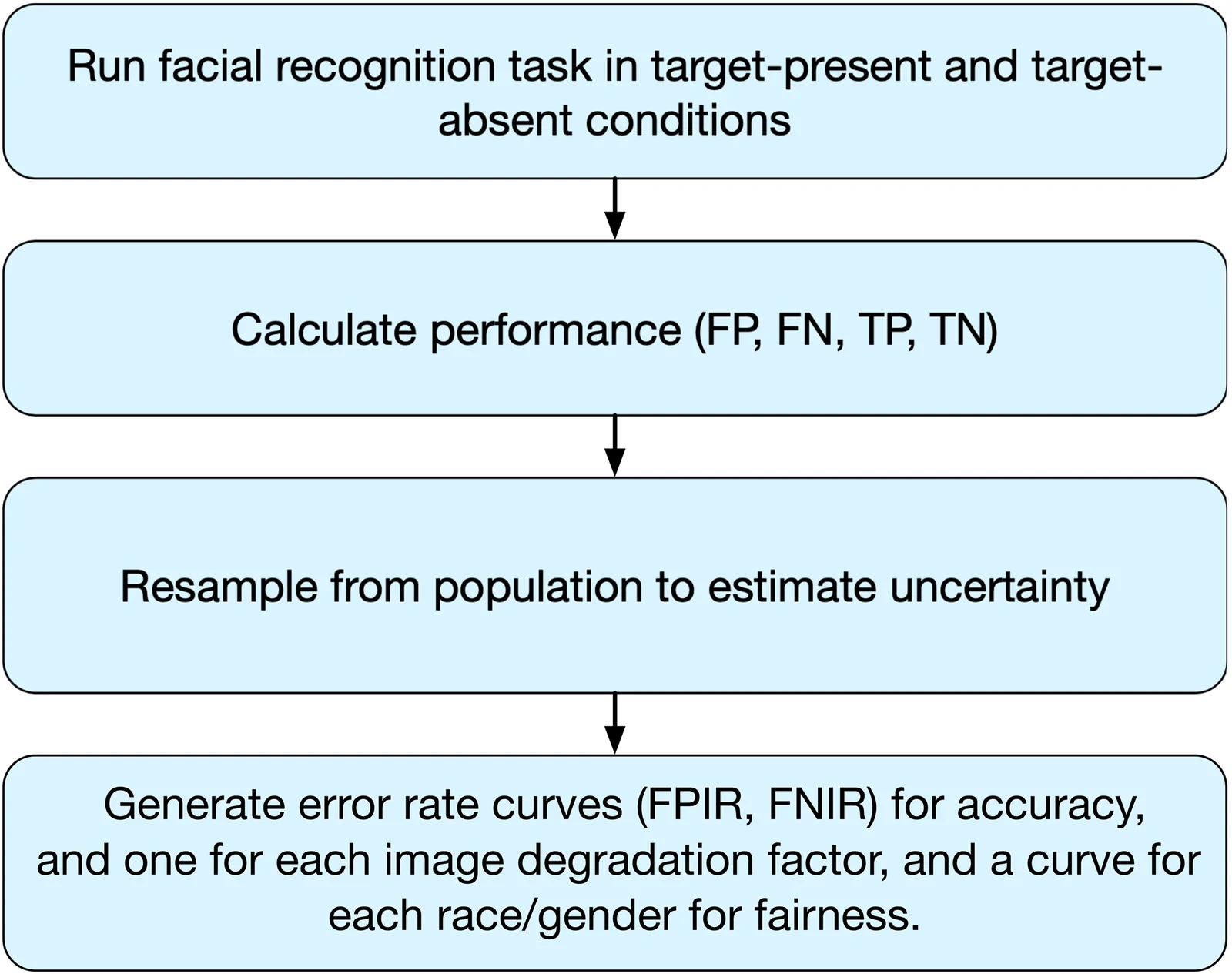
Our method to generate the conditional error-rate curves.

We selected the DeepFace/ArcFace framework because it is widely used, publicly available, computationally efficient, and has demonstrated strong performance in NIST FRVT evaluations [4], making it suitable for reproducible evaluation under realistic investigative conditions.

### 3.2 Error rates of 1:n identification task

We perform a total of 2^8^ = 256 replications for the simulation studies. For every treated face, we perform a 1-to-167 facial recognition task using DeepFace with the ArcFace model [64]. For the facial recognition outputs resulting from each of the 1-to-167 comparisons, we count the number of true positives, true negatives, false positives, and false negatives as follows: For the target-absent group, a match is counted as a false positive, and a non-match is counted as a true negative. For the target-present group, a correct match is counted as a true positive, an incorrect match is counted as a false positive. Similarly, a correct non-match is counted as a true negative, and an incorrect non-match is counted as a false negative.

After performing the facial recognition tasks for all replications following the above steps, we compute the number of false positives, false negatives, true positives, and true negatives for each replication. For each replication task, we calculate the false positive identification rate (FPIR) as,


$$\begin{array}{c} \text{FPIR}(N,T)=\frac{\begin{array}{c} \text{Num. non-mate searches where one or more enrolled candidates are}\\ \text{returned with distance at or below distance threshold} \end{array}}{\text{Num. non-mate searches attempted}} \end{array}$$ (1)

and the false negative identification rate (FNIR) as,


$$\begin{array}{c} \text{FNIR}(N,R,T)=\frac{\begin{array}{c} \text{Num. mate searches with enrolled mate found outside top R ranks or }\\ \text{distance above distance threshold} \end{array}}{\text{Num. mate searches attempted}} \end{array}$$ (2)

The facial recognition literature ([4]) distinguishes between error rates in “verification” tasks (1:1 image comparisons) and “identification” tasks (1:n search, when a system tries to identify a person from a gallery, e.g., police using a photo to search a database). Instead of a false non-match rate (FNMR), it uses FNIR (False Negative Identification Rate), the probability that the system fails to identify a person who is in the database. And instead of a false match rate (FMR), it uses a FPIR (False Positive Identification Rate), the probability that the system identifies a person who is not in the database.

The notation FNIR(*N*, *R*, *T*) includes three key parameters. *N* refers to the gallery size, or the number of identities stored in the enrollment database. *R* denotes the number of candidates returned–often called the rank or top-*R*–meaning the system returns the top *R* matches, and identification is considered successful if the correct identity appears among them. *T* represents the distance threshold used to determine whether a candidate is returned as a match; this value may be fixed or varied to analyze system performance across different operating points.

We use a fixed *N* and *T* in this study because this is akin to what law enforcement officers will likely do as they use FRTs. *N* is selected with our sample size calculation and *T* is set by the recommendation of the developers of the FRT algorithm we use. We do not impose a fixed top-R cutoff; instead, for each image, all gallery images with a cosine distance less than or equal to T are returned as matches. This mirrors a common law enforcement practice, where officers review all candidates above a certain confidence level rather than a fixed number of top matches. Note that lower cosine distance indicates greater similarity between faces.

### 3.3 Fairness

In this study, we analyze fairness by comparing error rates across race and gender groups. However, we do not employ more advanced fairness metrics or causal frameworks such as those proposed in prior work (e.g., [21–24]), which offer deeper insights into the mechanisms driving disparate performance.

## 4 Results

### 4.1 Accuracy

Fig 5 shows the results for evaluating the accuracy of the FRT algorithm. It displays the false positive identification rates (FPIRs) and false negative identification rates (FNIRs) of the algorithm, conditional on the image degradation factor. There is a curve for each of the factors: contrast, brightness, blur strength, pose, and resolution. The *x*-axis shows a normalized parameter value (with the baseline at 0). Distance from zero represents the magnitude of the manipulation, with 0 corresponding to the baseline (i.e., no degradation) and sign indicating direction where applicable. The axis ranges from −1.0 to 1.0. The *y*-axis shows the FPIR and FNIR values. The results in Fig 5 are consistent with NIST evaluations of facial recognition algorithms on 1:n identifications, which show higher FPIR under more permissive thresholds [17,67,68]. In this study, the high observed FPIR reflects that the developer-recommended threshold is relatively permissive, allowing more matches to be returned for target-absent searches and increasing the chance that a search produces at least one false positive.

**Fig 5:**
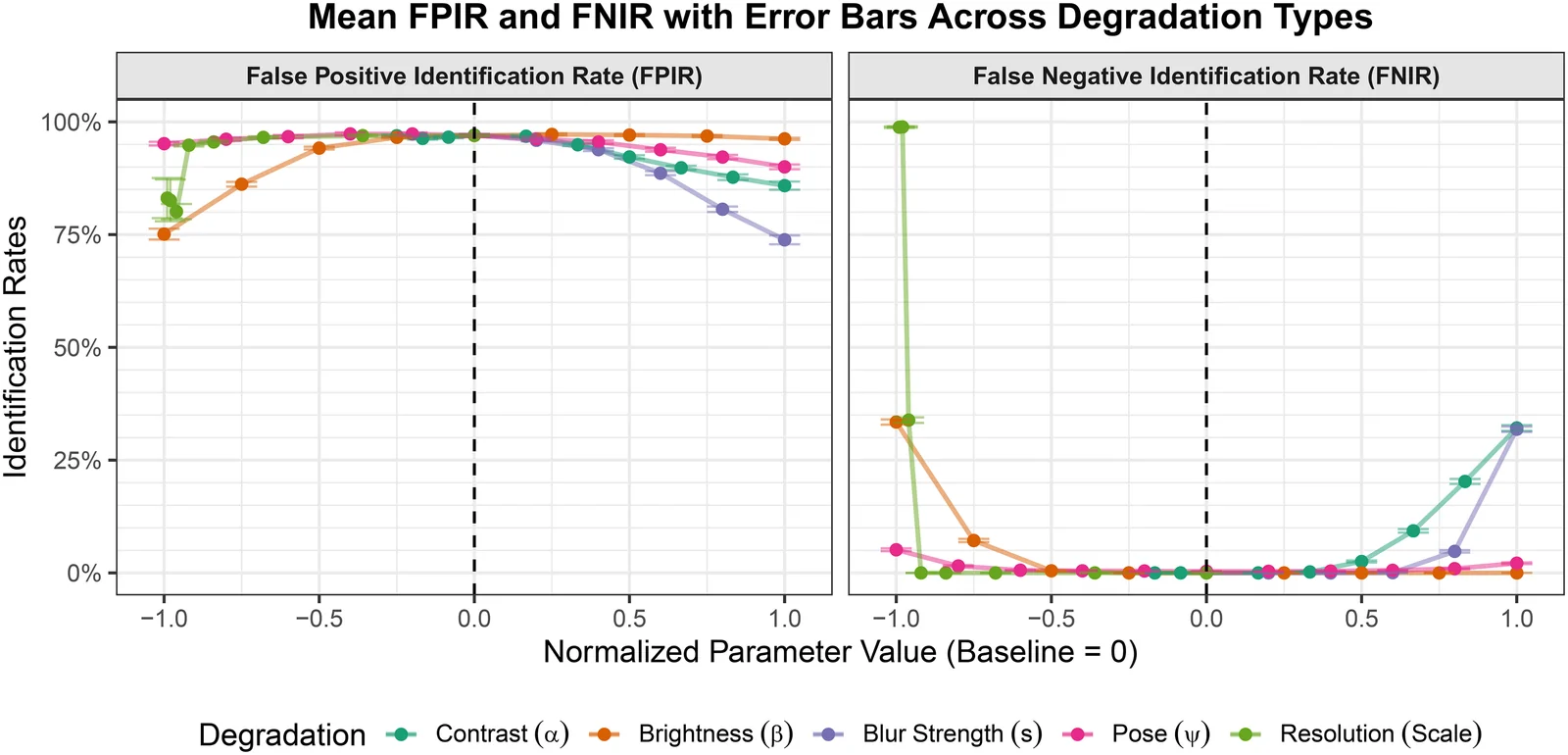
Overall conditional error-rate curves. False positive and false negative identification rates for different image degradation factors. The error bars denote the 95% confidence intervals obtained by resampling from the large population. The vertical dashed line represents the baseline image level.

The algorithm produces the most false positives when images are near their original, high-quality state, likely because both true and false matches yield higher similarity scores. As degradation increases, these spurious similarities diminish, leading to a drop in FPIR. This inverted U-shaped pattern suggests that degradation acts as a kind of natural filter, suppressing unintended similarity between different identities.

FPIR is particularly sensitive to high blur strength and low brightness, with resolution also producing substantial changes in FPIR. In contrast, pose has a comparatively smaller effect on FPIR.

For the false negative identification rate (FNIR), as image quality declines, the FNIR increases. Notably, reduced resolution has the most pronounced effect, indicating that the algorithm is more likely to fail to recognize a true match under these conditions. In contrast, changes in brightness, contrast, and blur have smaller but still sizeable impacts on FNIR, whereas pose has a comparatively smaller effect. This raises concerns about the reliability of FRT in law enforcement settings, where low resolution and high blur images are common.

We note that pose requires a separate discussion. The reason that it can be altered gradually, at such a massive scale, and with such low cost, is because we are using synthetic data. Since many police images of suspects show faces that are not directly facing the camera, accounting for pose variation is essential for understanding error rates in real-world conditions.

### 4.2 Fairness

Fig 6 presents how the conditional error rates vary across race and gender groups. Across all degradation types, there are clear disparities across race and gender groups. For instance, FPIR tends to be higher for Black individuals, particularly Black females, under many degradation conditions. This agrees with the literature on racial disparities in facial recognition technology ([17]). White males often have the lowest FPIR. These disparities grow less pronounced as degradation intensifies.

**Fig 6:**
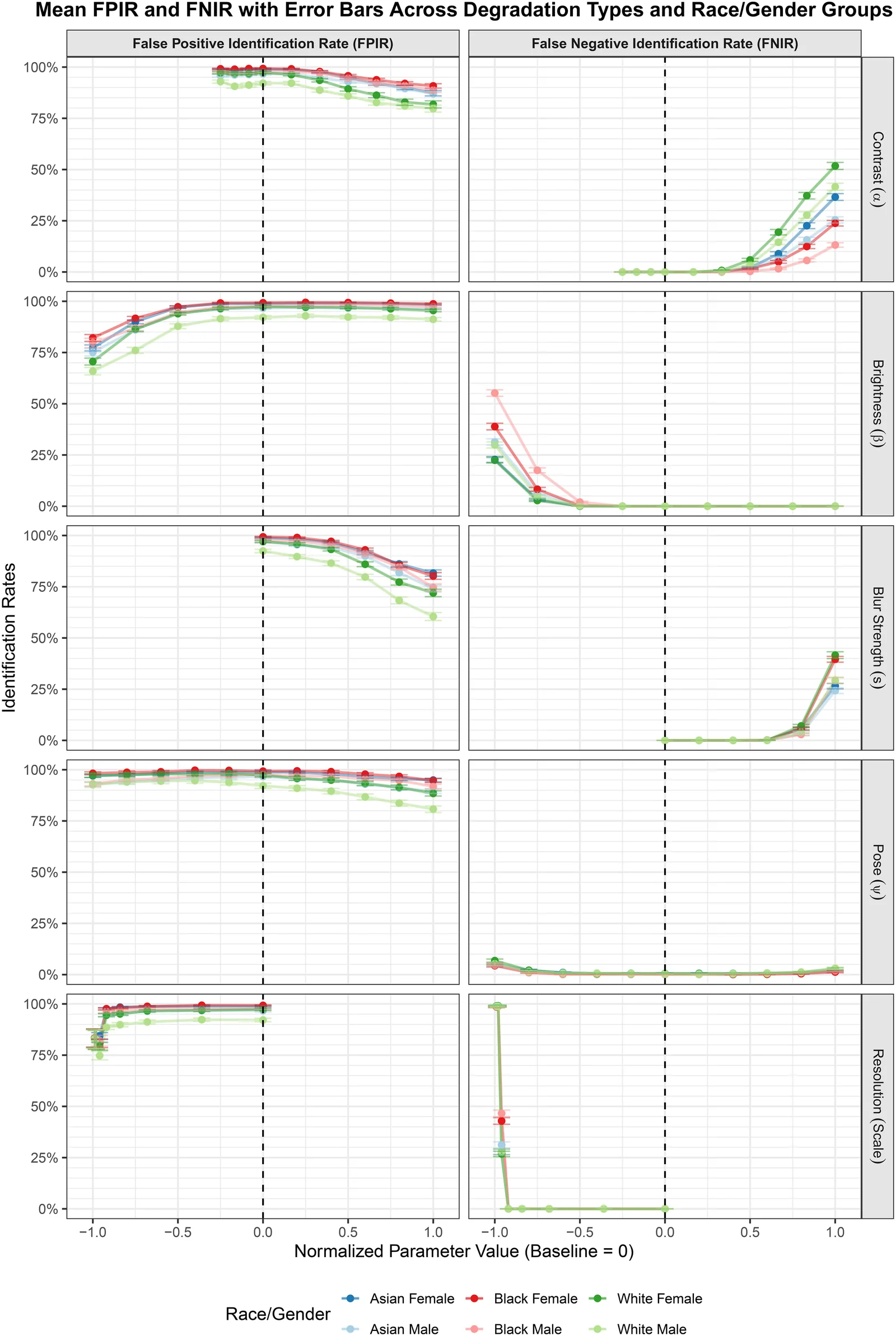
Demographic-specific conditional error-rate curves by race and gender. False positive and false negative identification rates for different image degradation factors. The error bars denote the 95% confidence intervals obtained by resampling from the large population. The vertical dashed line represents the baseline image level.

The corresponding plots show how FNIR varies across the same degradation types and demographic groups. The disparities here are notable: FNIR varies substantially across race and gender groups, and the groups experiencing the highest error rates differ across degradation conditions. For instance, White females exhibit particularly high FNIRs under contrast and blur degradation, while Black males exhibit relatively high FNIRs under low brightness. Resolution degradation produces especially large increases in FNIR across all groups, but the effects are unevenly distributed, with Black and Asian individuals disproportionately affected. In this case, the disparities grow more pronounced as degradation intensifies.

## 5 Discussion

Our results have several implications for the evaluation and operational use of facial recognition technology (FRT). First, the findings suggest that FRT performance should not be summarized using a single overall accuracy estimate. Instead, error rates depend on image quality conditions, particularly blur, brightness, contrast, and reduced resolution. This has important implications for law enforcement applications, where surveillance images are often degraded and operational conditions differ substantially from benchmark testing environments. The conditional error-rate curves developed in this study provide one possible framework for characterizing how performance changes across varying image conditions. The FPIR pattern observed here should be interpreted as specific to the operating conditions of this study. Prior 1:n studies have found that increased blur and reduced resolution can increase false-positive identification propensity [15], and more recent work has shown that combined probe and gallery degradation can substantially increase rank-1 FPIR [16]. Those studies differ from ours in gallery composition and size, thresholds, degradation procedures, and error-rate definitions, limiting direct comparison. Collectively, these results indicate that the relationship between image degradation and FPIR depends on the operational conditions under which the system is evaluated. Importantly, the two error types do not improve or worsen in the same direction. We find that image degradation generally increases FNIR while decreasing FPIR, changing the balance between failing to identify an enrolled individual and incorrectly returning a candidate when the individual is absent from the gallery. Thus, poorer image quality does not uniformly increase error; it changes the balance between two different types of errors. Whether a degraded image is suitable for a database search therefore cannot be determined from either FPIR or FNIR alone, but requires considering how both types of errors matter in the intended use of the FRT, and the consequences of each type of error.

For example, a police officer might notice that an image of an individual of questioned identity has low brightness and high blur. She could then consult the conditional error-rate curves in Fig 5 to determine how the error profile differs from that for a high-quality image; for example, FNIR increases substantially under some degradation conditions while FPIR decreases. A future research direction for this project is to design an algorithm that quantifies the amount of contrast, brightness, blur, pose, and resolution, and gives an estimated false positive and false negative rate for an image of this quality.

Second, our results highlight the importance of distinguishing between algorithmic performance and operational performance in practice. Benchmark evaluations typically assess algorithms under standardized testing conditions, whereas real-world investigative settings involve additional sources of variability, including image preprocessing, user decisions, threshold selection, and database construction. Accordingly, even highly accurate algorithms may produce unreliable or biased outcomes if deployed improperly or interpreted without consideration of image quality limitations.

Third, although we report differential error rates across demographic groups, we do not attempt a comprehensive evaluation of competing formal definitions of algorithmic fairness. Our goal is instead to characterize how accuracy changes across demographic groups under controlled image degradation conditions. Future work could extend these analyses by applying alternative fairness metrics, evaluating additional demographic characteristics, and studying how operational decisions influence disparate impacts in practice.

This study also has several limitations. First, the analyses rely on synthetic faces and controlled image manipulations, which may not fully capture the complexity of real-world surveillance imagery. Second, we evaluate a single implementation framework and facial recognition pipeline, and performance may differ across algorithms, thresholds, and enrollment databases. Third, our analysis focuses on degradation factors individually, whereas operational images often involve multiple simultaneous sources of degradation. Future work should therefore examine combined degradation effects, broader operational workflows, and human–algorithm interactions under realistic investigative conditions.

Nevertheless, synthetic data provide important methodological advantages by permitting controlled manipulation of image-quality factors while maintaining known ground truth identities. This framework enables systematic estimation of conditional false positive and false negative rates across varying image conditions and demographic groups. As FRT systems continue to evolve rapidly and become more widely deployed in criminal investigations, ongoing empirical validation under realistic operational conditions remains essential.

## 6 Conclusion

This study evaluated the effects of image degradation on the accuracy and fairness of facial recognition technology in 1:n identification tasks relevant to criminal investigations. Using synthetic faces, we systematically examined how blur, pose, brightness, contrast, and resolution influence false positive and false negative identification rates across demographic groups. In addition to estimating overall accuracy, we provide conditional error-rate curves showing how performance changes as image degradation increases across multiple image-quality factors and demographic groups.

Our results show that some forms of image degradation have substantially larger effects on performance than others. Reduced resolution has the largest effect on FNIR, while resolution, blur, brightness, and contrast also produce substantial changes in FPIR. Changes in blur, brightness, and contrast additionally produce sizeable increases in FNIR, but their effects are considerably smaller than that of reduced resolution. We also observe differential accuracy across demographic groups: FPIR is often higher for Black individuals, particularly Black women, whereas the demographic groups experiencing the highest FNIR vary across degradation conditions. Benchmark evaluations [4] often report low error rates under controlled, high-quality image conditions, but these estimates may not reflect performance in degraded surveillance imagery commonly encountered in practice. Together, these findings highlight the importance of evaluating FRT systems under realistic operational conditions rather than relying solely on summary benchmark accuracy estimates.

## Appendix

### A Factors for image degradation

#### A.1 Contrast and brightness.

Mathematically, an image can be represented by a rectangular matrix. For instance, an image of size M×N is a matrix with *M* rows and *N* columns, and the entries of the matrix are usually called pixels. In Python’s cv2 module, contrast and brightness are controlled by two separate parameters, α>0 and β, respectively. Given an input image *f*, the editing on each pixel *f*(*i*,*j*) using convertScaleAbs is regulated by the relationship,


$$\begin{array}{c} g(i,j)=\min\{255,|\alpha f(i,j)+\beta |\}, \end{array}$$

where *g*(*i*,*j*) denotes the (*i*,*j*)-th pixel of the output image. The function first applies an affine transformation to each pixel, then takes the absolute value, and finally clamps the result to the 8-bit range, i.e., all values are restricted to the range [0,255]. Within the affine transformation αf(i,j)+β, varying α results in a uniform scaling of the pixel values, which serves as the contrast parameter, whereas varying β results in a uniform translation of the pixel values, which serves as the brightness parameter. The affine transformation should be understood as the operation preceding the absolute value and saturation operations. In particular, for negative values of αf(i,j)+β, the absolute value operation reflects the values about 0, while values exceeding 255 are saturated at 255. Consequently, the overall transformation is not equivalent to a simple scaling and translation of the input pixel intensities in general.

Consider a 2×2 input image *f* given by


$$\begin{array}{c} f=(\begin{array}{cc} f(1,1) & f(1,2)\\ f(2,1) & f(2,2) \end{array})=(\begin{array}{cc} \min\{255,|1\times f(1,1)+0|\} & \min\{255,|1\times f(1,2)+0|\}\\ \min\{255,|1\times f(2,1)+0|\} & \min\{255,|1\times f(2,2)+0|\} \end{array})=(\begin{array}{cc} 12 & 24\\ 36 & 48 \end{array}). \end{array}$$

In other words, an input image always corresponds to the initial set of parameters (α,β)=(1,0). Now, if we change the contrast parameter α to 2 and the brightness parameter β to 50, the output image *g* is given by


$$\begin{array}{c} g=(\begin{array}{cc} g(1,1) & g(1,2)\\ g(2,1) & g(2,2) \end{array})=(\begin{array}{cc} \min\{255,|2\times f(1,1)+50|\} & \min\{255,|2\times f(1,2)+50|\}\\ \min\{255,|2\times f(2,1)+50|\} & \min\{255,|2\times f(2,2)+50|\} \end{array})=(\begin{array}{cc} 74 & 98\\ 122 & 146 \end{array}). \end{array}$$

To study the marginal effect of the contrast parameter, we consider the pairings


$$\begin{array}{c} \left(\alpha ,\beta )\in \{(0.25,0),(0.5,0),(0.75,0),(1,0),(1.5,0),(2,0),(2.5,0),(3,0),(3.5,0),(4,0)\right\}, \end{array}$$ (3)

i.e., we only vary the contrast parameter α but the brightness parameter β=0 remains unchanged. Similarly, to study the marginal effect of the brightness parameter, we consider the pairings $\begin{array}{c} \left(\alpha ,\beta )\in \{(1,-100),(1,-75),(1,-50),(1,-25),(1,0),(1,25),(1,50),(1,75),(1,100)\right\} \end{array}$, i.e., we only vary the brightness parameter β but the contrast parameter α=1 remains unchanged. In particular, the pair (α,β)=(1,0) results in no editing, thus representing the baseline.

Alternative non-linear are available. For instance, [69] provides an approach to replicate the GNU Image Manipulation Program’s non-linear in Python. For simplicity, we utilize the parametrized transformations contrast/brightness adjustments provided by the cv2 module, as implemented by convertScaleAbs.

#### A.2 Motion blur.

While degradations in blur come in multiple forms, we focus our discussion on motion blur because it is widely known as one of the most common blurs encountered in image processing. Contextually, police departments often perform facial recognitions based on images extracted from cameras or footage, in which motion blur is a recurrent phenomenon.

Motion blurs are simulated on an input face by applying a 2D convolution with a specifically designed kernel used to mimic the effect of linear movement during image capture, so that the pixel values are adjusted with a pre-specified angle and blur strength *s*. In this study, we focus on horizontal motion blurs, meaning the angle is always fixed at $0^{\circ }$. We study the marginal effect of horizontal motion blurs by considering different blur strength conditions s∈{0,20,40,60,80,100}, and *s* = 0 represents the baseline as it results in no motion blurs. Here, $\begin{array}{c} s \end{array}$ specifies the size of the motion blur kernel and serves as the blur strength condition. For the baseline case s=0, the original image is used directly without applying the motion blur treatment.

For an input face, we simulate motion blurs on it by applying a 2D convolution with a kernel defined in the following way.

For *s* > 0, let $\begin{array}{c} k \end{array}$ be an $\begin{array}{c} s\times s \end{array}$ matrix, defined by


$$\begin{array}{c} k_{i,j}=\left\{ \begin{array}{cc} 1,\; & \text{if }i=\lfloor \frac{s+1}{2}\rfloor ,\\ 0,\; & \text{otherwise}, \end{array}\right. \end{array}$$

that is, only the center row is set to ones, forming a horizontal line segment. The kernel is then normalized so that the sum of all its elements is 1:


$$\begin{array}{c} k_{i,j}\leftarrow \frac{k_{i,j}}{\sum_{i,j}k_{i,j}}. \end{array}$$

The motion-blurred image *g*(*x*,*y*) is produced by convolving the input image *f*(*x*,*y*) with the kernel *k*:


$$\begin{array}{c} g(x,y)=\sum_{i=1}^{s}\;\sum_{j=1}^{s}f(x+i-i_{0},y+j-j_{0})k_{i,j},\;i_{0}=j_{0}=\lfloor \frac{s+1}{2}\rfloor \end{array}$$

In practical terms, this means sliding the kernel over the image and, at each position, computing a weighted average of the corresponding neighboring input pixels, with the non-zero weights lying along the horizontal direction. Additionally, convolution with an image often involves flipping the kernel horizontally and vertically before applying it to the image, in our case, flipping the kernel horizontally has no effect because the non-zero weights are identical along the horizontal direction.

For illustration, consider a 3×3 kernel. For an interior pixel, the convolution can be written as


$$\begin{array}{c} g(x,y)=\sum_{i=-1}^{1}\sum_{j=-1}^{1}f(x+i,y+j)k_{i+2,j+2}. \end{array}$$

#### A.3 Pose.

We use the “restyle_e4e_ffhq” model for pose editing. Each image is first aligned and cropped to match the encoder’s training distribution. We then compute landmark-based transformations to record the geometric mapping between the original and aligned images. Next, we invert the aligned image into the StyleGAN3 latent space using the encoder, and run inference to obtain the latent code and a reconstructed image. Finally, we edit the latent code by moving it along a precomputed pose direction and synthesize the edited image. The landmark-based transformations are used to map the edited result back to the original pose and orientation, ensuring edits are consistent with the input image’s geometry. During the pose editing process, some images raised exceptions and could not be processed. These problematic images were excluded from the analysis. We study the marginal effect of pose by considering different parameters ψ=−5,−4,...,4,5, and ψ=0 represents the baseline as it results in no pose editing. However, we note that since the same procedures are performed even for ψ=0, the “baseline” images are slightly different from the original generated images. We adjust for this in our plots by adding the difference between the ψ=0 error rate for pose and the baseline error rate for the other image degradation factors. We add this fixed value to all the error rates and thus shift the pose curve upward for a more honest comparison. This shift is applied uniformly across pose conditions.

#### A.4 Resolution.

Image resolution refers to the number of distinct pixels used to represent a picture. Reducing resolution therefore discards high-frequency detail, and, even if an image is later enlarged, those lost details cannot be fully recovered. To mimic the low‑resolution CCTV or social‑media photographs that law‑enforcement officers often submit for facial searches, we apply the following two‑step process to every synthetic face:

1. **Down‑sample.** Shrink the original image by a user‑specified scale factor. We use Python’s cv2 INTER_AREA interpolation, which averages neighboring pixels and, therefore, best approximates a true reduction in optical resolution.
2. **Up‑sample.** Enlarge the low‑resolution image back to its original width *W* and height *H* with INTER_NEAREST interpolation. This preserves the blocky artifacts humans associate with low‑quality footage.

We study the marginal effect of resolution by considering different scales, namely, 1%, 2%, 4%, 8%, 16%, 32%, 64%, 100%, and scale = 100% represents the baseline as it results in no scaling.
